# Assessing research productivity in addiction datasets using OpenAlex

**DOI:** 10.1371/journal.pone.0339653

**Published:** 2026-02-02

**Authors:** David Melero-Fuentes, Cristina Rius, Rut Lucas-Domínguez, Juan Carlos Valderrama-Zurián

**Affiliations:** 1 Adolescence, Addictions and Development Research Group, Faculty of Psychology, Catholic University of Valencia, Valencia, Spain; 2 UISYS Group, Department of History of Science and Information Science, Faculty of Medicine and Dentistry, University of Valencia, Valencia, Spain; 3 Unit associated with the Interuniversity Institute for Advanced Research on the Evaluation of Science and the University (INAECU) UC3M-UAM, Madrid, Spain; 4 Spanish National Centre for Cardiovascular Research (CNIC), Madrid, Spain; 5 CIBERCV, Madrid, Spain; 6 CIBERONC, Valencia, Spain; Max Planck Institute for Solid State Research, GERMANY

## Abstract

The Open Science model has fostered new communication and archiving processes, supported by information technology. Within this framework, the open deposit of health-related datasets and their use in evaluating researchers and institutions are encouraged, with emerging information systems offering open alternatives to traditional databases. This study aims to analyse OpenAlex as a tool for retrieving research-derived datasets in a pilot study in the field of addictive substances, to determine whether it serves to automatically evaluate researchers and institutions inherent in the retrieved datasets. Results show that four repositories accounted for 85.8% of the 2782 datasets related to the addictive substances, being 30% of the records properly datasets followed by Comments on scientific investigation (21.4%), Monographs (15.1%) and Periodical publications (7.6%). In addition, missing information such as author identification (30.2%) or affiliation (69%) in the source repositories and data aggregators have been detected. Consequently, the assessment of researchers and institutions through datasets retrieved by OpenAlex would be improved by subsequently curating the information records. In conclusion, OpenAlex is a powerful tool in the Open Science medical ecosystem, and its accuracy could be enhanced by verifying the datasets collected via research information management infrastructures as well as by training professionals.

## Introduction

In recent decades, information technologies and the Open Science movement have changed the model of scientific communication [1,2]. Scientific journals have moved from being published on paper to being electronic, from being accessible by subscription to publishing articles in open access, either green or gold road [3,4], and from being published in journals to being deposited in repositories and available to open review [5].

In parallel with these developments, the open availability of datasets associated with research has been promoted [6]. A dataset is an organized collection of data, often grouped or tabulated, consisting of data collected through fieldwork, observations, research simulations, among others [7,8]. The types of files that contain datasets include spreadsheets (csv, ods, xlsx), text (xml, odf, doc, pdf), images (tiff, png, jpg), video (avi, ogv), and audio (opus, ogg).

Opening data is fundamental to the advancement of science because of the potential value of reuse (saving both time and resources), because it serves to reproduce and validate scientific findings, which is essential to the integrity of science, and because it allows researchers to develop and test new hypotheses and to develop and validate new data.

For researchers, the process of open data sharing involves two sets of circumstances:

Firstly, the guidelines based on four core desiderata-the FAIR Guiding Principles-and a subsequent evolution of them [9], as well as numerous subsequent efforts by some institutions to promote regulations that encourage good practices and principles in the deposit of datasets and quality, understood as the ease of retrieval and reuse of the data they contain [10]. Various initiatives have been added to these principles (FAIR), such as the Data Management Plan specified in the European Union’s H2020 mandate for research data [11]. Moreover, the Open Research Data Management Policy is required by Horizon Europe [12], which proposes planning for data processing and management, and from the United States, the 21st Century Cures Act (“the Act”) into law defines the creation of “information commons” to facilitate the open and responsible sharing of genomic and other data for clinical and research purposes [13,14].

Secondly, data repositories that allow us to deposit, store and manage datasets at different levels, such as adding new data in different versions, deleting data or depositing different types of files in the same dataset. Thus, we can find discipline-specific repositories such as Gene Expression Omnibus (https://www.ncbi.nlm.nih.gov/geo) and National Addiction & HIV Data Archive Program (NAHDAP) (https://www.icpsr.umich.edu/web/pages/NAHDAP/index.html); and generalist repositories such as FigShare (http://figshare.com), Dryad (https://datadryad.org), and Zenodo (http://zenodo.org).

Within this framework of Open Science, new bibliographic databases have been developed by exploiting the potential of interoperability of information systems and provide the scientific community and society with access to bibliographic references in an open format and/or by subscription, which differentiates them from the traditional databases (Web of Science (WoS) or Scopus) that have expanded their coverage or documentary types [15]. New information systems include open research systems like OpenAIRE (openaire.eu), Lens (lens.org), Dimensions (dimensions.ai) and OpenAlex (openalex.org) [16].

A difference between classical databases and these new databases lies in the processes used to index scholarly literature and manage bibliographic metadata. OpenAlex exemplifies this shift by mitigating geographic and linguistic bias through more balanced coverage than proprietary systems like WoS, although its less consistent metadata requires careful scrutiny despite its substantial potential for more representative analyses [17]. Thus, the coverage of WoS and Scopus is based on the indexing of documents, mainly scientific journal articles, selected journal by journal (in other words, inch by inch).Thus, the coverage of WoS and Scopus is based on the indexing of documents, mainly scientific journal articles, selected journal by journal (in other words, inch by inch), while these new databases work on the model of big data, artificial intelligence, machine learning and open information [18–21]. As far as transparency in their working methods allows, we know that they use APIs or metadata such as the DOI [Digital Object Identifier] [22] and the URI [Uniform Resource Identifier] [23] to integrate records from the various open databases they use as sources, such as DataCite and Crossref.

In particular, the coverage systems of these new databases are similar. The Open Access Infrastructure for Research in Europe (OpenAIRE) covers all university repositories in Europe and part of the Zenodo repository. Dimensions covers scientific literature, patents, archives of funded research projects, research-derived policies and research-derived data. For its part, OpenAlex is an open platform that emerged as an evolution of the Microsoft Academic Graph after its closure in 2021 [24]. Developed by Our Research, a non-profit organisation known for creating open-source tools for the academic community, OpenAlex seeks to fill the gap left by Microsoft Academic by providing an accessible, open-source index of scholarly works, authors, institutions, funding sources, and other fields that can establish itself as a competitor to WoS and Scopus [25]. OpenAlex has a broad multidisciplinary coverage (journal articles, books, datasets, and thesis), superior to other scholarly data sources such as Crossref, Dimensions, WoSCC and Scopus (https://openalex.org/about#comparison). OpenAlex most important data sources from which metadata are acquired, among others, Crossref, ORCID, PubMed, as well as “subject-area and institutional repositories from arXiv to Zenodo and many in between” [26,27]. For all these reasons, OpenAlex represents a platform with a high probability of being used for the evaluation of the scientific activity of research personnel and institutions. However, some previous studies have noted differences in the citations collected [25], as well as various limitations in the country assignment of authors’ institutions [18], missing institutions [28,29], and shortcomings in the curation of publication and documentary typologies [30].

Certain universities, such as the Sorbonne [31,32], are already using OpenAlex as an open resource (as opposed to WoS or Scopus platforms, which require a subscription for consultation of records deposited in repositories). According to their official statement, footnote 4, OpenAlex will be adopted as an important data source in the new version of the CWTS Leiden Ranking, which provides important insights into the scientific performance of over 1400 major universities worldwide [28,33].

These changes in the scientific ecosystem are also influencing the evaluation of research, considering not only journal articles, patents, books or book chapters, but also data, methodologies, computer programs or machine learning models. In this context, as highted in January 2022, the Coalition for Advancing Research Assessment (CoARA) [34] initiated a process to develop an agreement to reform research assessment. This agreement was signed on 15 July 2024 by 761 organizations from Europe, South America and Africa. The first point of the agreement mentions the recognition of the diversity of research contributions and academic stages according to the needs and nature of the research. In order to carry out this evaluation, it is recommended that a qualitative assessment be made, and metrics be used rationally. This transformation also implies that datasets and other non-traditional outputs must be findable, citable, and reliably attributable to authors and institutions, as required by responsible evaluation frameworks. Consequently, robust open infrastructures become essential, since they provide the metadata and traceability needed to retrieve, link, and evaluate diverse research contributions [25,35,36].

In this context, the area of research in addictive behaviours is characterised by a scientific production that covers several scientific fields (Social Sciences, Biomedicine,...) and is related to multiple disciplines (neurosciences, genetics, social work, psychology,...) with interest in data archiving following the guidelines established in the FAIR principles [9], as demonstrated by the creation of several initiatives (National Addiction & HIV Data Archive Program (https://www.icpsr.umich.edu/web/pages/NAHDAP/index.html); European Union Drugs Agency Data Home (https://www.euda.europa.eu/data_en); National Institute on Alcohol Abuse and Alcoholism Data Archive (https://nda.nih.gov/niaaa); National Institute on Drug Abuse Data Share (https://datashare.nida.nih.gov/); National Institute on Drug Abuse Center for Genetic Studies (https://nidagenetics.org/)). In this respect, OpenAlex is a tool of interest for the indexing and evaluation of datasets on addictive behaviours.

Furthermore, to our knowledge, studies on datasets in addiction are scarce and have focused only on raw data in substance abuse scientific journals [37] and on describing a drug abuse clinical trials web data share project [38].

In this way, taking into account the implementation of OpenAlex and the evaluation of datasets in a pilot study in the field of addictive substances, the present work aims to i) analyse the usefulness of OpenAlex as a tool for retrieving research-derived datasets; ii) perform a quantitative and qualitative characterization of the datasets retrieved; iii) examine whether an evaluation of researchers and institutions can be performed automatically from the retrieved datasets; iv) analyse document typology of files identified as ‘dataset’ resource type; and v) show similarities in the records.

## Methodology

### Search strategy and dataset

In May 2024, a bibliographic search was performed in OpenAlex with the equation: “cocaine OR cannabis OR heroin” in the Fulltext field and filtered in the Type field by dataset. A total of 2782 records were retrieved. The metadata of the records were downloaded as comma-separated values (csv) and tabulated in an.xlsx file.

The study sample was selected based on the expertise of the authors of the present work, who are part of a research group on addictive disorders and have been responsible for the Documentation Center on Drug Dependence and other Addictive Disorders website (cendocbogani.org) for more than 20 years.

### Characterization of the datasets

**Fig 1:**
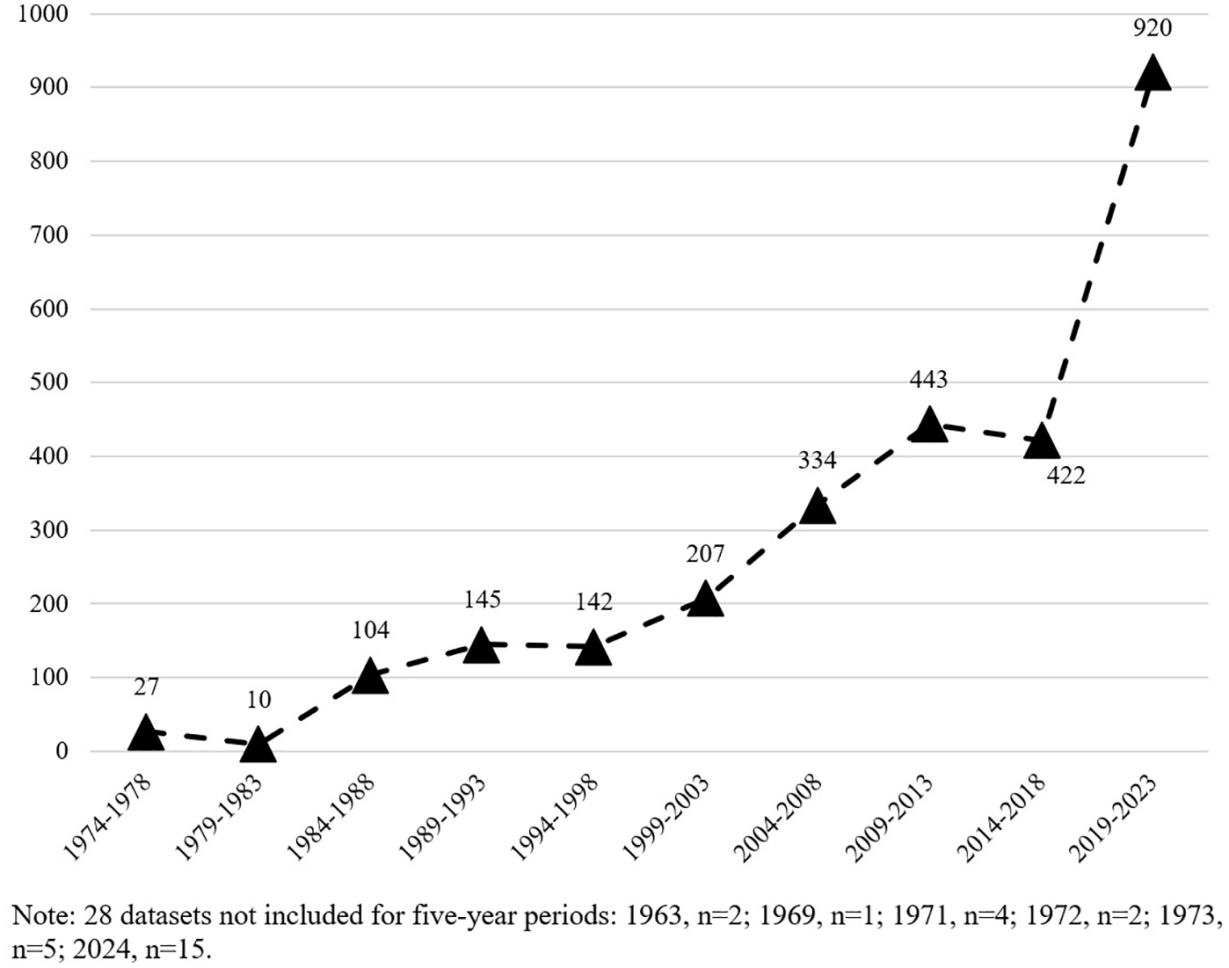
Chronological evolution of dissemination of datasets by five-year period (1974-2023).

A frequency analysis was performed on the qualitative variables, publication_date (1963–2024) and primary_location_source_display_name (SN). These analyses are shown in Fig 1 and Tables 1 and 2.

**Table 1:** Diachronic occurrence of dataset collection.

5-years period	datasets (n)			
	Crossref	Crossref|PubMed	DataCite	null
1974-1978	8	19		
1979-1983	7	3		
1984-1988	43	61		
1989-1993	72	72		1
1994-1998	79	63		
1999-2003	204	3		
2004-2008	330	4		
2009-2013	424	2	13	4
2014-2018	233	1	159	29
2019-2023	218		681	21
All years*	**1637**	**228**	**861**	**56**

*1963–2024 period.

**Table 2:** Occurrence of records by type of repository.

Repository	Type of repository	datasets (n)	datasets (%)
PsycEXTRA Dataset	specialised	997	35.84
Faculty Opinions – Post-Publication Peer Review of the Biomedical Literature	specialised	582	20.92
GNIF Global Biodiversity Information Facility	specialised	434	15.60
Figshare	generalist	373	13.40
Zenodo (CERN European Organization for Nuclear Research)	generalist	75	2.70
PsycTESTS Dataset	specialised	68	2.44
ISRCTN registry	specialised	46	1.65
Authorea	generalist	41	1.47
DATA.GOV	generalist	24	0.86
ICPSR Data Holdings	specialised	19	0.68
PeerJ preprints	specialised	13	0.47
The SHAFR Guide Online	specialised	11	0.40
AEA Randomized Controlled Trials	specialised	9	0.32
CABI Compendium	specialised	9	0.32
Human Rights Documents online	specialised	8	0.29
AAAS Articles DO Group	generalist	7	0.25
Supplementum Epigraphicum Graecum	specialised	7	0.25
Brill	generalist	6	0.22
Oxford Bibliographies Online Datasets	specialised	6	0.22
Default Digital Object Group	specialised	5	0.18
News Digital Object Group	specialised	5	0.18
F1000 - Post-publication peer review of the biomedical literature	generalist	4	0.14
Forefront Group	specialised	4	0.14
DataCite commons	generalist	4	0.14
Center of Alcohol and substance use studies	specialised	3	0.11
DataverseNL	generalist	3	0.11
AccessScience	generalist	2	0.07
DigiNole: Fsu’s digital repository	specialised	2	0.07
SciVee	specialised	2	0.07
American Heart Association Journals	specialised	2	0.08
Climate Change and Law Collection	specialised	1	0.04
Leader Mag Digital Object Group	specialised	1	0.04
ORMS Multimedia Group	specialised	1	0.04
RadioGraphics	specialised	1	0.04
Radiology Intelligent Assistant	specialised	1	0.04
Research Data Repository, Duke University	generalist	1	0.04
Science	generalist	1	0.04
Stroke	specialised	1	0.04
Africa health Research Institute (AHRI) Data Repository	specialised	1	0.04
Apollo Univerity of Cambridge	generalist	1	0.04
Autralian Catholic University	generalist	1	0.04

Similarly, the sample was described through the frequency of subject classifications, using the variables called concepts_display_name and topics_display_name (this analysis can be found in S1 and S2 Tables).

To determine the completeness of authorship and institution, a dichotomous analysis (yes/no) was performed on the occurrence of content in the author_names (AN) and author_institution_names (IN) fields (this analysis is shown in Table 3). Table 4 shows this association for each SN.

**Table 3:** Author-institution contingency.

		Institution (IN)		Total
		Yes	No	
**Author (AN)**	**Yes**	863 (31%)	1080 (38.8%)	1943 (69.8%)
	**No**	0 (0%)	839 (30.2%)	839 (30.2%)
**Total**		863 (31%)	1919 (69%)	2782 (100%)

Note: Pearson’s Chi-square test was statistically significant for co-occurrence associations and marginal probabilities (χ² = 540,234241; fd = 1; p < .000).

**Table 4:** Author-institution contingency by repository.

Source Name (SN)	Author null Institucion null		Author display Institucion null		Author display Institucion display	
	*n*	%	*n*	%	*n*	%
AAAS Articles DO Group	5	71.4%	2	28.6%		
AccessScience	2	100.0%				
AEA Randomized Controlled Trials			9	100.0%		
Africa health Research Institute (AHRI) Data Repository			1	100.0%		
American Heart Association Journals	1	20.0%	4	80%		
Apollo Univerity of Cambridge			1	100.0%		
Authorea			28	68.3%	13	31.7%
Autralian Catholic University			1	100.0%		
Brill	5	83.3%	1	16.7%		
CABI Compendium	9	100.0%				
Center of Alcohol and substance use studies	3	100.0%				
Climate Change and Law Collection	1	100.0%				
DATA.GOV	24	100.0%				
DataCite commons			4	100.0%		
DataverseNL			3	100.0%		
Default Digital Object Group	5	100.0%				
DigiNole: Fsu’s digital repository	1	50.0%			1	50.0%
F1000 - Post-publication peer review of the biomedical literature			2	50.0%	2	50.0%
Faculty Opinions – Post-Publication Peer Review of the Biomedical Literature			9	1.5%	573	98.5%
Figshare			318	85.25%	55	14.75%
Forefront Group	3	75.0%	1	25.0%		
GNIF Global Biodiversity Information Facility	434	100.0%				
Human Rights Documents online	7	87.5%	1	12.5%		
ICPSR Data Holdings	6	31.6%	13	68.4%		
ISRCTN registry			46	100.0%		
Leader Mag Digital Object Group			1	100.0%		
News Digital Object Group	1	20.0%	4	80.0%		
ORMS Multimedia Group	1	100.0%				
Oxford Bibliographies Online Datasets			5	83.3%	1	16.7%
PeerJ preprints	13	100.0%				
PsycEXTRA Dataset	300	30.1%	544	54.6%	153	15.3%
PsycTESTS Dataset			68	100.0%		
RadioGraphics					1	100.0%
Radiology Intelligent Assistant			1	100.0%		
Research Data Repository, Duke University					1	100.0%
Science			1	100.0%		
SciVee	1	50.0%	1	50.0%		
Stroke	1	100.0%				
Supplementum Epigraphicum Graecum	7	100.0%				
The SHAFR Guide Online	8	72.7%	2	18.2%	1	9.1%
Zenodo (CERN European Organization for Nuclear Research)			13	17.3%	62	82.7%

Note: Percentage calculated by rows.

### Documentary typology of the files included in the registers

The evaluation of the documentary typology of the files included in the retrieved records was carried out by means of a stratified random analysis of datasets (n = 771; 27.7% of the records), weighted by the records of each repository (n = 41). Selection was based on a 95% confidence level (z = 1.96) and a sampling error of ±3%. Access to the dataset from OpenAlex and to the file hosted in the repository was then manually examined by all authors. In this way, the original sources were accessed and through manual verification the documentary typologies were classified. This analysis is presented in Table 5.

**Table 5:** Typologies of documents retrieved as “datasets” and occurrence of repositories.

Type	Dataset (n)	%	Repositories (n)	Most occurring repositories in this typology	Most occurring repository (n)	Most occurring repository (%)
Article	5	0.65%	3	Figshare	3	60%
Clinical Trial	13	1.69%	1	ISRCTN registry	13	100%
Comments on a scientific investigation	165	21.40%	4	Faculty Opinions – Post-Publication Peer Review of the Biomedical Literature	161	97.58%
Dataset	231	29.96%	10	GNIF Global Biodiversity Information Facility/ Figshare/ Zenodo	120/72/ 19	91.34%
Interview	1	0.13%	1	PsycEXTRA Dataset	1	100%
Factsheet	12	1.56%	1	PsycEXTRA Dataset	13	100%
Graph	23	2.98%	2	Figshare	22	95.65%
Guide	1	0.13%	1	The SHAFR Guide Online	1	100%
Meeting abstract	8	1.04%	1	PsycEXTRA Dataset	17	100%
Meeting slides	7	0.91%	1	PsycEXTRA Dataset	7	100%
Monograph	116	15.05%	1	PsycEXTRA Dataset	116	100%
News	13	1.69%	4	PsycEXTRA Dataset	8	61.54%
Preprint	8	1.04%	1	AEA Randomized Controlled Trials	8	100%
Periodical publication	59	7.65%	2	PsycEXTRA Dataset	48	96%
Report	66	8.56%	5	PsycEXTRA Dataset	53	81.54%
Test	11	1.43%	1	PsycTESTS Dataset	11	100%
Thesis	1	0.13%	1	DigiNole: Fsu’s digital repository	1	100%
Video/Podcast	9	1.17%	2	PsycEXTRA Dataset	8	88.89%
[Link error]	22	2.85%	11	Figshare/ PsycEXTRA Dataset	4/ 4	36.36%

### Similarities in the records

To identify possible similarities between records, the DOI and display_name metadata were examined. Thus, (a) records with the same DOI were identified using the logical function IF and; (b) records with identical titles or with small differences in characters (upper/lower case, punctuation) were manually reviewed and the analysis was performed by three professionals with expertise in health sciences and scientific documentation (CR, JCVZ, RLD) to determine why identical titles occurred. This analysis is presented in Table 6.

**Table 6:** Title similarities.

Type of similarity	similarities (n)	∑ records (*%*)
Two records	132	264 (9.49)
≥ two records	33	572 (20.56)
**Total**	165	836 (30.05)

## Results & discussion

### Characterization of the datasets

A total of 2782 records classified as datasets in 41 different repositories were obtained, belonging mainly to the subject classifications (named concepts_display_name) of Psychology, Medicine and Psychiatry and (named topics_display_name) “Endocannabinoid System and Its Effects on Health”, “Neurobiological Mechanisms of Drug Addiction and Depression” and “Epidemiology and Interventions for Substance Use” (S1 and S2 Tables). The diachronic evolution of the sample is distributed between 1963 (2 records) and 2024 (15 records). There is an upward trend per quinquennium in the number of records classified as datasets. Only two quinquennia have fewer records than the previous one (1979–1983 and 2014–2018). The changes between the quinquennia average a difference of 99.22 records (SD 159.25). Percentage-wise, three periods can be distinguished, (a) the first two quinquennia accumulate a percentage of less than 1% of the records, (b) the period of quinquennia between 1984 and 2003 presents percentages of records between 3% and 8%, and (c) the last four quinquennia show percentages of 12% and 15% in the first three quinquennia and an increase to 33.07% (1:3) in the last quinquennium (Fig 1).

The results show that there is an increase in the deposit of datasets over the years, which has a point of ascent from the five-year period 1999–2003, coinciding with the 3B of Open Access [39,40], and another more notorious peak from 2014–2018, coinciding with the implementation of Open Access in relation to the funding and regulation of open data, both in Europe with the 2020 horizon and other international policies [13].

Regarding the source where the bibliographic reference of the dataset is indexed and through which it has been included in OpenAlex, it is observed that Crossref provides 58.84% of the datasets (n = 1637), mainly in the last five quinquennia (n = 1409) (Table 1). Second, the largest indexing of bibliographic references to datasets comes from DataCite, which provides 25% of the total datasets for the period 2019–2023 (n = 681). Both indexes contribute 89.79% of the datasets (n = 2498) (Table 1).

In the context of research information management infrastructure, Crossref [41], launched in 2000, complements the coverage of other widely used commercial sources such as WoS and Scopus, while PubMed dominates among open databases in the health sciences [42].

One of the advantages of Crossref, OpenCitations, DataCite, OpenAIRE and OpenAlex is that they offer a wide range of metadata for any purpose free of charge, without limiting the maximum amount of metadata that can be retrieved [43].

Furthermore, OpenAlex, is popular because it has an even greater documentary reach than the main databases that are its main sources (Crossref and Microsoft Academic Graph) [42]. The results obtained in this study, which place CrossRef as the main repository for addictive substances datasets, are consistent with the fact that CrossRef has established itself as one of the preferred open metadata repositories [16].

In second place is DataCite, which contains records from almost 3,000 data repositories [44,45]. However, the temporal coverage of DataCite, which started in 2009, is inferior to that of Crossref, which, unlike the former, retrieves data back to its creation date (2000).

Analysis of the variable primary_location_source_display_name, which refers to the repositories (n = 41) where the dataset is hosted, shows that the PsycEXTRA dataset is the repository with the highest number of datasets (n = 997; 35.84%), followed by Faculty Opinions – Post-Publication Peer Review of the Biomedical Literature (n = 582; 20.92%), GNIF Global Biodiversity Information Facility (GNIF) (n = 434; 15.6%) and Figshare (n = 373; 13.40%). These four repositories provide 85.77% of the records (n = 2386) (Table 2).

As expected, among the diversity of repositories found, the PsycEXTRA Dataset stands out, which is a specific repository on psychology and behavioural sciences created by the American Psychological Association [46], closely related to our field of study, drugs. In this sense, 6 of the top 10 repositories are thematic, highlighting PsycExtra and PsycTESTS Dataset, Faculty Opinions – Post-Publication Peer Review of the Biomedical Literature, GNIF Global Biodiversity Information Facility, ISRCTN Registry of clinical trials, or ICPSR Data Holdings, while the rest are general repositories with multidisciplinary themes such as Figshare, Zenodo, Authorea, DATA.GOV. Overall, 65.85% (n = 27) are specialised repositories and 34.15% (n = 14) are generalist repositories. Specialised repositories account for 80.3% of the datasets while generalist repositories account for 19.7% of the datasets. These results are in line with previous studies [45] confirming that the authors of the datasets, may have a preference for certain specialized repositories over more generalist ones, following the recommendations of some publishers who encourage the deposit of data in discipline-specific repositories [47]. With respect to generalist repositories, there is a dispersal of datasets across the wide variety of non-specialist repositories, which contributes to the difficulty of locating and reusing datasets.

Analysis of the AN variable shows that of the 2782 records retrieved on the topic of addictive substances, 839 (30.2%) did not provide authorship information (Table 3). Of these, 434 were from the GNIF Global Biodiversity Information Facility repository, 309 were from the PsycExtra repository, and 105 were distributed among 21 repositories (Table 4).

Regarding the variable author_institution_names, 1919 (69%) had an empty field (Table 3). Of these, the largest distributions are in the PsycEXTRA dataset (n = 844), GNIF Global Biodiversity Information Facility (n = 434), and Figshare (n = 318). The remaining empty fields (n = 327) in the IN metadata are distributed across 38 repositories (Table 4).

This problem with authorship and institutional affiliations has also been described by Krause & Mongeon [32]. In the present study, we have also observed how the contingency of the values of both variables (yes/no), the co-occurrence probabilities are in the range of 8.6 (between 30.2% and 38.8%). On the one hand, the probability of finding neither authors nor institutional affiliation is 30.2%, the probability of finding authors with affiliation is 31%, and the probability of finding author information without institutional affiliation is 38.8% (Table 3).

It has been reported that OpenAlex indexes more than 200 million authors through their ORCID or OpenAlex ID (OAID), and more than 100,000 institutions through their Research Organisation Register (ROR) or OAID [42]. However, in our study, despite the registration of an extensive network of affiliations, it has been observed that there are some gaps (omissions) in certain fields, which, firstly, makes it difficult to properly cite the document and, secondly, affects the citations estimation of this document and evaluation of its authors and institutions. This creates an even bigger problem, which extends to the systems for obtaining indicators fed by OpenAlex, and also affects the evaluation system of researchers who deposit their datasets to promote Open Science.

This can lead to inaccurate citation counts, which are important to suggest in a “*bug report or improvement suggestion*” to improve OpenAlex. Therefore, given the inaccuracy of citations that can result from these issues, it is advisable to use different bibliographic databases to verify the citation counts received for each dataset, to provide a more accurate picture of the research impact of datasets [42].

The results of the present study on the lack of identification of institutions in the datasets retrieved through OpenAlex are in line with those of Zhang et al. [30], who found that about 60% of the scientific journal articles in OpenAlex in the social sciences and humanities did not contain information on the institution [30]. In this respect, Ortega and Delgado-Quirós [16] postulate that the cause of the omissions may be due to the third-party sources from which they are taken, in this case OpenAlex, such as PubMed, DataCite or Crossref. Data on the author and/or institution may not be included in the metadata of the repository in which they are hosted or are difficult to access, as is the case with GNIF Global Biodiversity Information Facility (100% absence of author and institution labels). Moreover, the way in which each database and repository labels the Author, Institution and/or Cites fields can make proper retrieval difficult [16].

In contrast, Velez-Estevez et al. [43] indicate that OpenAlex, Dimensions and Scopus Affiliations are the most complete databases for identifying the institutions of publications, and ORCID, Scopus Authors and Publons (now integrated with Web of Science Research ID) for author-based analyses. In addition, one of the features that sets OpenAlex apart is that it allows author and affiliation information to be retrieved via the Research Organisation Register (ROR) ID by implementing this process [43].

However, as the RORs and the affiliationIdentifier, affiliationIdentifierScheme and schemeURI have only been integrated into Datacite in version 4.3 [48], and in Crossref until 2021 [49], it is possible that problems are currently creeping in with the identification (tagging) of author affiliation information of a dataset hosted in this collector for a period prior to this version.

At the same time, recent scholarly literature confirms the incompleteness of some information fields in the records hosted in the repositories, which is consistent with our results regarding the lack of institutions in the retrieved datasets, e.g., 85.25%, 17% and 68.4% in Figshare, Zenodo and ICPSR respectively (Table 4). Furthermore, in the case of ICPSR, 31.6% of the records were missing author and institution [45].

### Document typology of files identified as ‘Dataset’ resource type

The analysis of the random sample stratified by repository shows that 30% of the records retrieved are datasets, according to definition of dataset [9,10], deposited in 10 different repositories, representing 24% of the total number of repositories providing datasets (120 in GNIF, 72 in Figshare and 19 in Zenodo). Among the other most common document types, 21.4% were comments on scientific research (n = 165) in the Faculty Opinions repository, 15% were monographs and 8% were reports, both in the PsycExtra repository (Table 5).

Almost a quarter of the files analysed are ‘comments on research’, which is consistent with research by Johnston et al. [45], who in their study found records which, despite being described as ‘datasets’ in the metadata, are not. This is the case for the repository Faculty Opinions Ltd (now H1 Connect) (https://connect.h1.co/about), which does not contain any identifiable datasets per se, although it uses the term ‘dataset’ as a resource type for indexing in Crossref, as there is no other term in the resource type field that fits this type of document [45].

These results show that, although the sharing of data in the field of addiction has been encouraged by the creation of various institutional initiatives (U.S. NIH, NIDA), firstly, it becomes evident that there is a lack of knowledge of the concept of a dataset among the researchers who are responsible for depositing and appropriately tagging the metadata of the documents registered in the repositories. Secondly, the ambiguity between the records recovered under this document typology differs from other types of documents whose classification does not give rise to confusion (clinical trials, original articles). In turn, Scholarly Knowledge Graphs (SKG) systems would support and ameliorate publication workflows improving and guaranteeing metadata quality [50]. Furthermore, it would be highly recommended that all academic repositories implement the roadmap developed by the Repositories Expert Group in support of researchers as part of the Data Citation Implementation Pilot (DCIP) project, an initiative of FORCE11.org and the NIH-funded BioCADDIE project (https://biocaddie.org).

In parallel, data repositories would facilitate by one hand, the location, re-use and citation of data sets through an appropriate process of metadata creation with semantic enrichment [51], and on the other hand, by providing a metadata field clearly dedicated to associated publications [52]. The ultimate goal, is to ensure a correct repository of datasets with all required tags properly completed [53]. To date, 8.9% of the 41 repositories in this study have followed and committed to this roadmap [54].

Finally, one suggestion for OpenAlex to improve the collection of datasets would be to check the repositories and the information they collect through Crossref and Datacite. In the present study, 997 records retrieved from Crossref under the repository name PsycEXTRA dataset do not match the original name (APA PsycEXTRA).

On the other hand, it is important to remember that, as Velez-Estevez et al. [43] point out, open databases may contain more errors or less accuracy in some of their data, due to the cataloguing or indexing process, since the curation and pre-processing of a large amount of scientific data requires a great deal of effort and economic resources that non-profit organizations sometimes cannot afford.

### Similarities in the records

Of the 2782 records retrieved, a total of 48 records (1.73%) are duplicates, i.e., they appear twice with the same DOI and the same OpenAlex record id. Therefore, 24 OpenAlex records are duplicated in the search results. Furthermore, 165 title similarities were found in the datasets, of which 132 similarities occur in two records and 33 similarities occur in more than two records (Table 6).

The above results indicate an error in the collection of records after the bibliographic search, i.e., there are no duplicate records, but the 24 records are duplicated in the results grid provided by OpenAlex, as well as in the download of the data. On the other hand, a co-occurrence of title similarities is observed. This aspect may be related to the concentration of interest in some data, whether they are versions of the same data or a coincidence of thematic data, giving us an idea of the data with the greatest flow or interest. At this point it is relevant to consider good practices in the title given to the datasets, as it is the main description of the data and when searching for datasets a massive repetition of titles can be perceived as duplicity, so it would be highly recommended to provide more precise titles that disambiguate the datasets, adding unambiguity to each dataset.

The inaccuracy found is in line with previous studies. In the case of Gerasimov et al. [55], in a study focused on the citation of datasets by scientific papers, it can be observed how Crossref omits the DOI of access to the dataset, even in publications that reference datasets; most of these documents belonging to publications edited by Elsevier. On the other hand, Johnston et al. [45], attribute its origin to the fact that some repositories issue DOIs for each file, while others assign DOIs at the level of the study, e.g., Zenodo assigns a different DOI to each deposited dataset and also to each of its versions, then OpenAlex identifies each of these DOIs as an independent dataset record. The resulting dispersion leads to a significant lack of precision.

## Conclusion

OpenAlex is presented in the context of Open Science as an open access database with broad multidisciplinary coverage, highlighting its usefulness for the development of indicators of scientific activity and the evaluation of researchers’ and institutions’ curricula in terms of scientific production and data repository. The study suggests that research on the scientific literature on addictive substances datasets needs to consult more than one bibliographic source in order to fill the gaps identified due to the lack of metadata in some fields (author, institution). Moreover, this analysis provides a detailed description of issues to support the continuous improvement of OpenAlex, a scholarly data resource increasingly used and valued by researchers. In this regard, it would be advisable for the sources from which OpenAlex aggregates to implement the curation process of the records it indexes, in order to accurately identify and tag the datasets it hosts [43]. The lack of information in certain author and institution fields can lead to an underestimation of scientific output.

The evaluation of datasets produced by an author or institution cannot be done automatically from OpenAlex, as is traditionally done with Web of Science and Scopus for scientific articles, because 70% of the records are not datasets.

It is necessary to train researchers in how to fill in the bibliographic records of the repositories and the naming of document typologies to avoid indexing as a dataset works that are monographs or other types of documents. With proper curation by researchers before repositories, OpenAlex would be a more accurate source of datasets.

There is also the problem that the databases that feed OpenAlex incorrectly index some items as datasets, and consequently they are hosted under wrong type of document.

Finally, the existence of duplicates, the inclusion of all versions or even the appearance of an additional record, as in the case of Zenodo, can lead to an excessive evaluation of the records of a given author or institution, as well as the number of citations received, if no prior disambiguation is made and only a specific piece of data is evaluated.

However, we believe that this situation observed in OpenAlex can be corrected to improve the quality of the service offered to the scientific community. We note two actions that may have an impact on this improvement.

On the one hand, the European Open Science Cloud establishes an interoperability framework for the exchange of scientific data and calls for a standard data model and an export and exchange format that facilitates open access to scientific data regardless of the discipline to which it belongs, whose guidelines are followed by various research entities such as OpenAIRE, OpenAlex, Crossref of DataCite. This would make it possible to standardize the definition of metadata in a common data model [50].

On the other hand, regarding the specific field of addictive substances, as of January 25, 2023, the U.S. NIH Data Management Plan and Sharing Policy call for the sharing of study data. And in 2009 the National Institute on Drug Abuse itself established the addictions repository recognizing the value of data sharing [56]. In addition, it has generated a program that provides assistance to depositors and data seekers to ensure proper data sharing following FAIR principles [57].

## Limitations & future studies

The results obtained obey an exploratory study on addictive substances using three representative terms of this topic (cocaine, cannabis or heroin) [58] in the search equation executed in OpenAlex, so datasets hosted on other platforms may have been excluded. Therefore, comparisons with other scholarly data sources such as OpenAIRE, Dimensios or Scopus should be included in future studies. Furthermore, OpenAlex recently started to index datasets and their coverage of datasets is far from being complete.

## Supporting information

S1 Table Frequency of Topics in the retrieved datasets. (DOCX)

S2 Table Frequency of Concepts in the retrieved datasets. (DOCX)
