# Supplementary material for: Assessing research productivity in addiction datasets using OpenAlex
Source: PLoS One. 2026 Feb 2;21(2):e0339653. doi: 10.1371/journal.pone.0339653 (PMC12863530; doi:10.1371/journal.pone.0339653)
Supplement: S1 Table — (DOCX) [file pone.0339653.s001.docx]

**Supplemental Table S1.** Frequency of Topics in the retrieved datasets.

| **Topic** | **n** |
| --- | --- |
| Endocannabinoid System and Its Effects on Health | 428 |
| Neurobiological Mechanisms of Drug Addiction and Depression | 387 |
| Epidemiology and Interventions for Substance Use Disorders | 351 |
| Opioid Epidemic in the United States | 224 |
| Forensic Toxicology Methods and Analysis | 144 |
| Global Epidemiology of HIV and Drug Use | 90 |
| Effects of Prenatal Alcohol Exposure on Offspring | 83 |
| Attention-Deficit/Hyperactivity Disorder | 78 |
| Molecular Mechanisms of Depression Treatment Strategies | 72 |
| Geometric Processing of Remote Sensing Imagery | 71 |
| Emotion Regulation and Psychopathology in Children and Adolescents | 66 |
| Molecular Mechanisms of Synaptic Plasticity and Neurological Disorders | 64 |
| Structure and Function of G Protein-Coupled Receptors | 62 |
| Effects of Stress on Brain Function and Health | 55 |
| Neuroscience and Education: Bridging Research and Practice | 52 |
| Schizophrenia Research and Treatment | 50 |
| Gamma-Aminobutyric Acid Metabolism in Plants | 45 |
| Network Analysis of Psychopathology and Mental Disorders | 43 |
| Organized Crime and Criminal Networks Analysis | 41 |
| Impact of Homelessness on Health and Well-being | 39 |
| Role of Sigma Receptors in Cellular Signaling | 39 |
| Digital Mental Health Interventions and Efficacy | 36 |
| Impact of Religion and Spirituality on Health Outcomes | 34 |
| Therapeutic Potential of Psychedelic Therapy | 34 |
| Neuroimmune Interaction in Psychiatric Disorders | 33 |
| Polypharmacy and Inappropriate Medication Use in Elderly | 33 |
| Patterns and Impacts of Drug Use in Brazil | 32 |
| Analysis of Brain Functional Connectivity Networks | 31 |
| Developmental Origins of Adult Health and Disease | 31 |
| Epilepsy and Seizures | 31 |
| Effects of Ketogenic Diet on Health | 30 |
| Pathogenesis and Treatment of Alcoholic Liver Disease | 29 |
| Neural Mechanisms of Memory Formation and Spatial Navigation | 28 |
| Oxytocin and Social Behavior Regulation | 28 |
| Neural Mechanisms of Cognitive Control and Decision Making | 27 |
| Cognitive Mechanisms of Anxiety and Depression | 24 |
| Psychological Resilience and Well-being | 24 |
| Role of Neuropeptides in Physiology and Disease | 24 |
| Structure and Function of Nicotinic Receptors | 24 |
| Challenges in Pain Management and Treatment | 23 |
| Psychology of Heroism and Courage | 22 |
| Brown Adipose Tissue Function and Physiology | 21 |
| Computational Methods in Drug Discovery | 21 |
| Deep Brain Stimulation for Neurological Disorders | 21 |
| Neurobiological Mechanisms of Placebo and Nocebo Effects | 21 |
| Impact of Food Insecurity on Health Outcomes | 19 |
| Impact of Fructose on Metabolic Health | 19 |
| Sleep's Role in Memory Consolidation and Regulation | 19 |
| Effects of Beta-Adrenergic Agonists in Livestock | 18 |
| Epigenetic Modifications and Their Functional Implications | 18 |
| Management of Poisoning and Toxic Exposures | 18 |
| Positive Youth Development in the United States | 18 |
| Prenatal Exposure to Antiepileptic Drugs | 18 |
| School Absenteeism and Social Withdrawal Behavior | 18 |
| Social Media Use and Impact on Society | 18 |
| Elicitor Signal Transduction for Metabolite Production | 17 |
| Polyamines and Biogenic Amines in Biology and Health | 16 |
| Analysis of Electromyography Signal Processing | 15 |
| Developmental Pharmacology in Pediatric Drug Use | 15 |
| Impact of Digital Technology on Parenting and Education | 15 |
| Long-Term Effects of Testosterone on Health | 15 |
| Sensory Analysis in Food Science Research | 15 |
| Sweeteners' Taste and Impact on Health | 15 |
| Impact of Stigma on Mental Health Care | 14 |
| Production of Recombinant Pharmaceuticals in Plants | 14 |
| Theories of Behavior Change and Self-Regulation | 14 |
| Epidemiology and Management of Bipolar Disorder | 13 |
| Impact of Social Media on Well-being and Behavior | 13 |
| Mechanisms and Management of Neuropathic Pain | 13 |
| Prevention and Treatment of HIV/AIDS Infection | 13 |
| Role of Positive Emotions in Well-Being | 13 |
| Cyanogenic Glycosides in Plants and Industrial Wastewaters | 12 |
| Empowerment and Collaboration in Community Health | 12 |
| Impact of Mass Incarceration on Society and Individuals | 12 |
| Mental Health and Quality of Life | 12 |
| Metabolism and Function of Arachidonic Acid Derivatives | 12 |
| Neurotoxic Effects of Anesthetic Agents on Brain Development | 12 |
| Autism Spectrum Disorders | 10 |
| Eating Disorders and Body Image Concerns | 10 |
| Feeding Disorders in Children with Autism Spectrum Disorders | 10 |
| Global Impact of Sex Work and Trafficking | 10 |
| Mental Health of Refugees and Immigrants | 10 |
| Neural Mechanisms of Respiratory Control and Homeostasis | 10 |
| Sleep and Insomnia | 10 |
| Therapeutic Applications of Bee Venom Therapy | 10 |

Topics with fewer than 10 records are not shown.
