# Supplementary material for: Assessing research productivity in addiction datasets using OpenAlex
Source: PLoS One. 2026 Feb 2;21(2):e0339653. doi: 10.1371/journal.pone.0339653 (PMC12863530; doi:10.1371/journal.pone.0339653)
Supplement: S2 Table — (DOCX) [file pone.0339653.s002.docx]

**Supplemental Table S2.** Frequency of Concepts in the retrieved datasets.

| **Concept** | **n** |
| --- | --- |
| Psychology | 1615 |
| Medicine | 1354 |
| Psychiatry | 1176 |
| Computer science | 827 |
| Neuroscience | 554 |
| Biology | 529 |
| Internal medicine | 501 |
| Pharmacology | 467 |
| Cannabis | 453 |
| Drug | 442 |
| World Wide Web | 436 |
| Download | 413 |
| Addiction | 341 |
| Receptor | 309 |
| Heroin | 304 |
| Clinical psychology | 299 |
| Chemistry | 282 |
| Genetics | 215 |
| Biochemistry | 200 |
| Dopamine | 179 |
| Gene | 163 |
| Political science | 143 |
| Environmental health | 139 |
| Geography | 135 |
| Mathematics | 133 |
| Sociology | 120 |
| Law | 118 |
| Substance abuse | 118 |
| Business | 113 |
| Psychotherapist | 110 |
| Computational biology | 107 |
| Physics | 106 |
| Cocaine use | 98 |
| Social psychology | 97 |
| Nucleus accumbens | 93 |
| Cognition | 90 |
| Demography | 84 |
| Developmental psychology | 84 |
| Cognitive psychology | 83 |
| Pathology | 82 |
| Opioid | 79 |
| Anesthesia | 77 |
| Economics | 76 |
| Self-administration | 74 |
| Paleontology | 71 |
| Philosophy | 71 |
| Artificial intelligence | 68 |
| Family medicine | 66 |
| Programming language | 66 |
| Central nervous system | 65 |
| Statistics | 64 |
| Botany | 63 |
| Engineering | 63 |
| Quantum mechanics | 62 |
| Cocaine abuse | 61 |
| Drugs of abuse | 59 |
| Endocrinology | 57 |
| Population | 56 |
| Craving | 55 |
| Criminology | 55 |
| Crack cocaine | 52 |
| Administration (probate law) | 51 |
| Human immunodeficiency virus (HIV) | 50 |
| Cannabidiol | 48 |
| Cannabinoid | 48 |
| Heroin addiction | 48 |
| Pregnancy | 47 |
| Art | 46 |
| Dopaminergic | 44 |
| Substance use | 43 |
| Cell biology | 42 |
| Alternative medicine | 41 |
| Cannabis sativa | 41 |
| Computer security | 41 |
| Linguistics | 41 |
| Virology | 41 |
| Disease | 40 |
| Heroin dependence | 40 |

Concepts with fewer than 40 records are not shown.
